# Diphtheria outbreak, Northern Territory of Australia, 2025 to 2026

**DOI:** 10.2807/1560-7917.ES.2026.31.23.2600443

**Published:** 2026-06-11

**Authors:** Anthony DK Draper, Mark Sistrom, Kimberley McMahon, Robert Duguid, Jerry Chen, Georgia Cramp, Thomas Cherian, Mirsaed Miri Nargesi, Dzulfikaar Sutandar, Kevin Freeman, Kelly Lomas, Tarrant Tolotta, Jenine C Gunn, Rowena Boyd, Jann Hennessy, Tinus Creeper, Jaimee Harbidge, Fatima Sarmiento, Rebecca Curr, Jennifer Yan, Joshua R Francis, Robert Baird, Hayley Stratton, Jane Davies, Catherine S Marshall, Sonja Janson, Nerida Moore, Rebecca Wardell, Eugene Athan, George Drewett, Liz Moore, Elly Layton, Unni Susil Kumar, Rosalind Webby, Ashish Yadav, Vicki L Krause, Bhavi Ravindran, Ella M Meumann

**Affiliations:** 1 Centre for Disease Control, Northern Territory Government Department of Health, Darwin, Australia; 2 National Centre for Epidemiology and Population Health, Australian National University, Canberra, Australia; 3 Global and Tropical Health Division, Menzies School of Health Research, Charles Darwin University, Darwin, Australia; 4 Territory Pathology, Royal Darwin Hospital, Darwin, Australia; 5 Berrimah Veterinary Laboratory, Department of Agriculture and Fisheries, Northern Territory Government, Darwin, Australia; 6 Research Institute for the Environment and Livelihoods, Charles Darwin University, Darwin, Australia; 7 Paediatric Department, Royal Darwin Hospital, Darwin, Australia; 8 Health Statistics and Informatics, Northern Territory Department of Health, Darwin, Australia; 9 National Critical Care and Trauma Response Centre, Darwin, Australia; 10 Centre for Disease Control, Northern Territory Government Department of Health, Alice Springs, Australia; 11 Department of Infectious Diseases, Division of Medicine, Royal Darwin Hospital, Darwin, Australia; 12 Department of Infectious Diseases, Division of Medicine, Alice Springs Hospital, Alice Springs, Australia; 13 Aboriginal Medical Services Alliance Northern Territory, Darwin, Australia; * These authors contributed equally to this work and share first authorship.; ** These authors contributed equally to this work and share last authorship.

**Keywords:** Diphtheria, *Corynebacterium diphtheriae*, Northern Territory, Australia, Vaccination, Aboriginal Australians, outbreak, whole genome sequencing

## Abstract

Because of high vaccination rates, the Northern Territory of Australia had not seen locally transmitted toxigenic *Corynebacterium diphtheriae* since the 1990s, until cutaneous diphtheria cases emerged in May 2025 and respiratory cases in March 2026. By 30 April 2026, there had been 131 cases (34 respiratory, 97 cutaneous) in an ongoing outbreak characterised by a high proportion of cutaneous cases with transmission driven by crowding and consequent poor skin health. Most patients have had mild disease due to prior vaccination.

The Northern Territory (NT) of Australia spans 1.3 million km^2^ and has a population of 255,000, with 30% identifying as Aboriginal Australians, many of whom live in remote communities [1]. The NT had been free of locally acquired diphtheria disease since the late 1990s, until a cutaneous case was notified in May 2025. Since then, cutaneous and respiratory notifications have been increasing. We describe the current outbreak as at April 2026.

## Epidemiology of the outbreak

**Figure 1:**
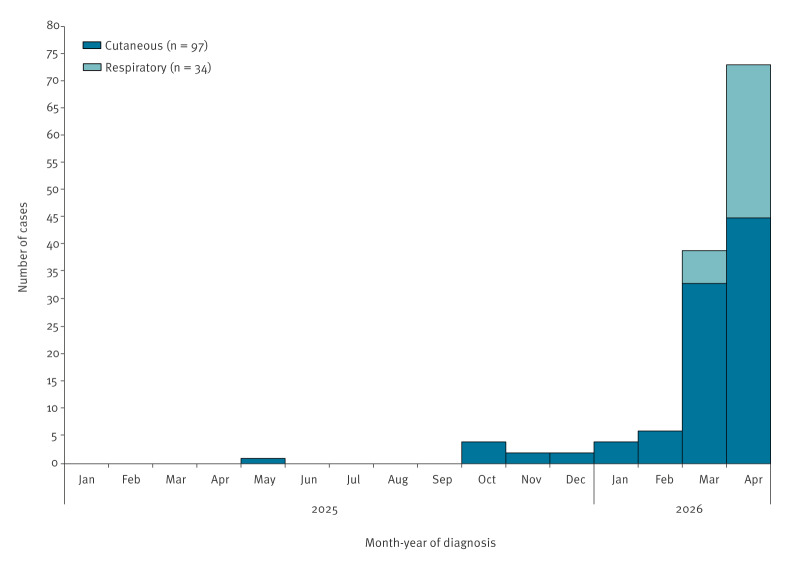
Epidemic curve of diphtheria cases, by presentation type and month of symptom onset, Northern Territory of Australia, 1 January 2025–30 April 2026 (n = 131)

Between 1 January 2025 and 30 April 2026, there were 131 notifications of diphtheria (34 respiratory and 97 cutaneous) in the NT (Figure 1). A confirmed case required isolation of toxigenic *Corynebacterium diphtheriae* (130 cases) or *Corynebacterium ulcerans* (1 case) from a clinically relevant site with clinical evidence of either an upper respiratory tract infection or skin lesion [2]. Case characteristics are presented in Table 1. Most (n = 125; 95%) notifications were in Aboriginal Australians. A larger number of respiratory cases were detected in the arid desert region of Central Australia (n = 24) than the tropical Top End region (n = 10), whereas more cutaneous cases were detected in the Top End region (n = 71) compared with Central Australia (n = 26). 

**Table 1:** Characteristics of diphtheria cases, Northern Territory of Australia, 1 Jan 2025–30 April 2026 (n = 131)

Characteristics	Respiratory (n = 34)		Cutaneous (n = 97)		Total (n = 131)	
	n	% of all respiratory	n	% of all cutaneous	n	% of all
**Indigenous status**						
Aboriginal	34	100	91	93.8	125	95.4
Non-Aboriginal	0	0	6	6.2	6	4.6
**Sex**						
Male	15	44.1	43	44.3	58	44.3
Female	19	55.9	54	55.7	73	55.7
**Location in Northern Territory**						
Top End	10	29.4	71	73.2	81	61.8
Central Australia	24	70.6	26	26.8	50	38.2
**Age group (years)**						
0–4	0	0	10	10.3	10	7.6
5–9	4	11.8	7	7.2	11	8.4
10–14	9	26.5	1	1	10	7.6
15–19	5	14.7	7	7.2	12	9.2
20–34	10	29.4	23	23.7	33	25.2
35–49	5	14.7	41	42.3	46	35.1
50–64	1	2.9	8	8.2	9	6.9
**Hospitalised**						
Yes	12	35.3	27	27.8	39	29.8
No	22	64.7	70	72.2	92	70.2
**Intensive care unit admission**						
Yes	2	5.9	0	0	2	1.5
No	32	94.1	97	100	129	98.5
**Died**						
Yes	1	2.9	0	0	1	0.8
No	33	97.1	97	100	130	99.2
**Time since last vaccination**						
Unvaccinated	2	5.9	6	6.2	8	6.1
< 1 year	0	0	19	19.6	19	14.5
1–4 years	9	26.5	29	29.9	38	29
5–9 years	8	23.5	19	19.6	27	20.6
≥ 10 years	15	44.1	24	24.7	39	29.8
**Number of vaccines**						
0 doses	2	5.9	6	6.2	8	6.1
1–2 doses	4	11.8	24	24.7	28	21.4
3–4 doses	10	29.4	38	39.2	48	36.6
≥ 5 doses	18	52.9	29	29.9	47	35.9
**Symptoms and complications (respiratory cases only)**						
Sore throat	29	85.3	NA			
Tonsillitis	18	52.9				
Fever	15	44.1				
Pharyngeal exudate	12	35.3				
Cough	10	29.4				
Pharyngitis	9	26.5				
Cervical lymphadenopathy	7	20.6				
Pseudomembrane^†^	3	8.8				
Myocarditis	1	2.9				
Shock	1	2.9				
Respiratory distress	1	2.9				
Neuropathy	0	0				
Renal injury	0	0				
**Treatment**						
Diphtheria antitoxin	3	8.8	0	0	3	2.3
Azithromycin – 7-day course	27	79.4	41	42.3	68	51.9
Azithromycin – single dose	3	8.8	7	7.2	10	7.6
L-A BICILLIN (benzathine benzylpenicillin tetrahydrate)	23	67.6	20	20.6	43	32.8

NA: not applicable.

Among the 34 respiratory cases, the median age was 18 years (range: 7–52), and 19 were female. One probable myocarditis-related death occurred in an adult who had received all childhood vaccines but no booster within the past 10 years. Toxin-positive *C. diphtheriae* was detected in skin and throat swabs of this patient post-mortem. The median time since last diphtheria-containing vaccine was 9.3 years (range: 1–23) for respiratory cases and 4.5 years (range: 0–49) for cutaneous cases. Of the 12 severe (hospitalised or died) respiratory diphtheria cases, two were unvaccinated, and seven of the remaining 10 had not received a diphtheria-containing vaccine in more than 10 years. 

## Laboratory aspects of the outbreak

Territory Pathology is the Government pathology provider for public hospitals in the NT, while private pathology providers serve primary care outside of the hospital setting. Here, we describe results from laboratory testing conducted by Territory Pathology during the outbreak; methods can be found in the Supplementary Material. 

**Figure 2:**
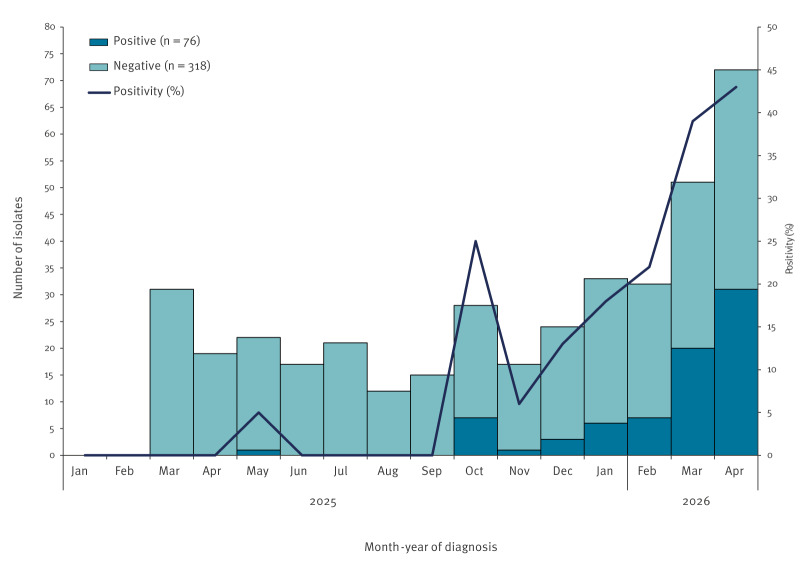
Cutaneous *Corynebacterium diphtheriae* isolates tested for the toxin gene by PCR, and proportion of positive tests, Northern Territory of Australia, 1 January 2025–30 April 2026 (n = 394)

Before this outbreak, isolation of non-toxigenic *C. diphtheriae* alongside beta-haemolytic streptococci and *Staphylococcus aureus* from purulent skin lesions was common in the NT. In two previous NT snapshot studies, no toxin gene-positive cutaneous *C. diphtheriae* isolates were found among 251 isolates from 2005 to 2011, and 41 isolates from 2022 [3,4]. From March 2025, Territory Pathology began systematically conducting PCR testing for the *tox* gene on *C. diphtheriae* culture isolates [5]. Of 67 wound swabs with toxin-positive *C. diphtheriae* isolated, 43 were from lower limbs, 16 from upper limbs, six from head/neck and two from torso/genital lesions. The proportion of *tox-*positive cutaneous *C. diphtheriae* culture isolates reached its peak in this report’s timeframe in April 2026 (Figure 2). Culture for other organisms was conducted on 64 of 67 skin swab cultures with toxin-positive *C. diphtheriae* isolated; all were polymicrobial, and the co-isolated pathogens included 51 *S. aureus* (14 with meticillin resistance), 45 Group A *Streptococcus*, 22 *Arcanobacterium haemolyticum*, eight Group C/G *Streptococcus*, 13 coliform bacteria, and 10 anaerobes. 

The graph reflects only the toxin tests done at Territory Pathology, and includes all cutaneous isolates tested including any duplicates from the same patient and body site.

Noting the potential range of clinical diphtheria presentations, Territory Pathology began in March 2026 setting up routine throat swab cultures for *C. diphtheriae* on Hoyle’s selective medium to improve detection of respiratory diphtheria. The *tox* gene was detected by PCR in 23 of 33 respiratory *C. diphtheriae* culture isolates tested in March and April 2026. Among 17 of the positive throat swabs that underwent culture for other organisms, six yielded Group A *Streptococcus* in addition to toxin-positive *C. diphtheriae*. 

Seventy-six cutaneous and respiratory isolates underwent antimicrobial susceptibility testing using gradient diffusion strips; all were susceptible to erythromycin and susceptible with increased exposure to penicillin (i.e. susceptible but at increased concentration) (Table 2).

**Table 2:** Antimicrobial susceptibility results for diphtheria outbreak isolates, Northern Territory of Australia, 1 Jan 2025–30 April 2026 (n = 76)

Antimicrobial	Number of isolates with MIC (μg/mL)								Clinical MIC breakpoints (μg/mL)	
	0.016	0.032	0.064	0.125	0.25	0.5	1	2	CLSI [26]	EUCAST [27]
Erythromycin	61	11	4	0	0	0	0	0	S ≤ 0.5, R > 1	S ≤ 0.06, R > 0.06
Penicillin	0	0	0	0	6	67	3	0	S ≤ 0.12, R > 2	S ≤ 0.001, R > 1
Doxycycline	0	0	2	36	14	3	0	0	S ≤ 4, R > 8	S ≤ 0.5, R > 0.5
Ciprofloxacin	0	0	33	1	0	0	0	0	S ≤ 1, R > 2	S ≤ 0.001, R > 0.5
Sulfamethoxazole-trimethoprim	0	0	0	0	2	2	42	6	S ≤ 2, R > 2	S ≤ 0.5, R > 0.5

CLSI: Clinical and Laboratory Standards Institute; EUCAST: European Committee on Antimicrobial Susceptibility Testing; MIC: minimum inhibitory concentration; R: resistant; S: susceptible.

Testing conducted using gradient diffusion strips. All 76 isolates were tested for the first line agents penicillin and erythromycin; a subset was tested against the other antimicrobials.

**Figure 3:**
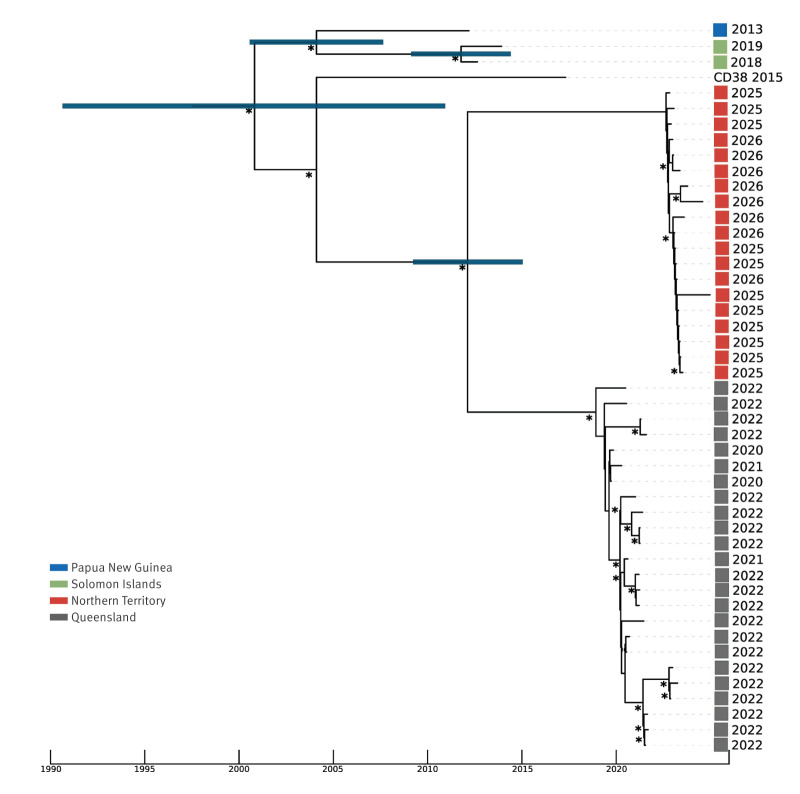
Maximum likelihood phylogeny of evolutionary relationships among toxin gene-bearing *Corynebacterium diphtheriae* ST381 isolates (n = 47)

Whole genome sequencing (WGS) was performed on 19 *tox*-positive cutaneous isolates; all belonged to sequence type (ST) 381 with a median pairwise difference of three single nucleotide polymorphisms (SNPs) (range: 0–19). The NT ST381 sequences were compared against publicly available ST381 sequences, including 24 sequences linked to an outbreak from 2020 to 2022 in the neighbouring Australian jurisdiction of Queensland, two sequences from the Solomon Islands and one from Papua New Guinea [6]. The NT sequences diverged from Queensland sequences by a median of 72 SNPs (range: 65–85). Time-calibrated phylogenetic analysis showed that the NT outbreak genomes formed a distinct clade from Queensland genomes (Figure 3), with the estimated most recent common ancestor dating to January 2017 (95% credible interval: June 2013–April 2019). No clinically relevant antimicrobial resistance markers were detected, and all 19 isolates were confirmed to carry *tox* gene allele type 20 [7]. National surveillance has confirmed that a dominant ST381 strain is responsible for cases in 2025 and 2026 in the NT, Western Australia, Queensland and South Australia [8]. 

CD38 was used as the reference genome [28]. Horizontal bars represent 95% credible intervals for node ages. 5,000 bootstrap replicates were conducted, and asterisks indicate nodes with bootstrap support > 95%. Branch lengths are proportional to time, with the time scale shown in years below the tree.

Phenotypic testing for toxin production using the Elek immunoprecipitation assay is not currently available in Australia. Five isolates were confirmed to be Elek-positive at an overseas public health laboratory, including the first NT detection, two respiratory isolates from patients who received diphtheria antitoxin (DAT), an isolate from the probable myocarditis-related death, and an additional cutaneous isolate.

## Public health response

In response to the respiratory diphtheria cases, the NT issued, on 25 March 2026, a Public Health Alert and published interim guidelines for the clinical management of diphtheria in primary care [9]. The strategy includes empiric antibiotic treatment of suspected cases (both cutaneous and respiratory), vaccination of under-vaccinated individuals (as per the Australian National Immunisation Program (NIP)), and contact tracing to identify further cases and to provide treatment. A population-based vaccination programme targeting under-vaccinated individuals and those who have not received a diphtheria-containing vaccine in the past 5 years is being implemented as a key control measure, as the majority (n = 84; 64%) of cases presented < 10 years since last vaccine [10,11].

## Discussion

Diphtheria is a vaccine-preventable disease caused by toxigenic corynebacteria, typically *C. diphtheriae* or less commonly *C. ulcerans*, resulting in cutaneous or respiratory presentations, or asymptomatic carriage [12]. Respiratory diphtheria can be life-threatening, resulting from local and systemic effects of diphtheria toxin, particularly in unvaccinated or under-vaccinated individuals, and in children aged < 5 years [13,14]. Pseudomembranous inflammation of the upper respiratory tract can cause respiratory obstruction, while systemic toxin effects may result in myocarditis and polyneuropathy [13]. Transmission occurs via respiratory droplets or contact with wound exudate, with an incubation period of 2–5 days [12]. Penicillin or macrolide antibiotics eradicate the organism and reduce transmissibility [15]. The only treatment that is effective against diphtheria toxin is DAT, and it is indicated primarily for respiratory infections [16].

Diphtheria incidence in Australia has been low since vaccination programmes were introduced in the 1930s, with most cases in returned travellers [12]. The Australian NIP recommends diphtheria-toxoid vaccines at 2, 4, 6 and 18 months, and 4 years, with a booster at 12–13 years. There is also a maternal vaccination programme where diphtheria-containing vaccines are used [10]. A diphtheria toxoid vaccine booster is recommended (but not NIP-funded) in adults at 50 years of age, every 10 years for travellers to countries where health services are difficult to access, or every 5 years for areas with high transmission [10]. As at February 2026, 95.4% of Aboriginal and/or Torres Strait Islander children and 91.9% of all children in the NT at age 5 years had received all recommended diphtheria-toxoid vaccine doses [11].

Despite high vaccination rates, diphtheria has re-emerged in the NT. Disease has been predominantly mild. Sore throat and tonsillitis have been the most common respiratory features and only three cases in the reporting period had a pseudomembrane. Skin lesions have been uniformly polymicrobial, with co-isolation of *S. aureus* and/or beta-haemolytic streptococci in all lesions at Territory Pathology; while such lesions are a reservoir for onward transmission, the role of cutaneous toxigenic *C. diphtheriae* as a pathogen in the context of recent vaccination and the presence of the other organisms is uncertain. There has been limited use of DAT due to initial limited availability and subsequent recommendation for use only in those with a pseudomembrane.

The NT outbreak has predominantly affected Aboriginal Australians and follows an outbreak of 29 infections caused by ST381 *C. diphtheriae* in Far North Queensland from 2020 to 2022, which also predominantly affected Aboriginal and/or Torres Strait Islander peoples [6,17]. Due to inequities which contribute to socioeconomic disadvantage, Aboriginal Australians disproportionately experience crowded and substandard housing and high rates of homelessness, and a greater overall burden of disease [18]. Diphtheria is well known to take hold in marginalised populations, including displaced persons and people experiencing homelessness [19]. The high rates of scabies, pyoderma, invasive Group A streptococcal infection, acute rheumatic fever and rheumatic heart disease reflect conditions conducive to spread of skin infection [18,20], and it is no surprise that introduction of a toxin-positive *C. diphtheriae* strain in the NT has resulted in rapid spread.

The strategy for control of the outbreak in the NT has been similar to that used in the United Kingdom [21] and has included contact tracing with prophylactic antibiotics and booster vaccination within 12 months for high- and medium-risk contacts. Empiric treatment of suspected cutaneous and respiratory diphtheria has been employed to interrupt transmission chains. We must acknowledge key recommendations from recent diphtheria outbreaks across Europe [22-24]: Routine immunisation programmes must be strengthened and boosters offered, especially to at-risk populations. Collaboration with Aboriginal Community Controlled Health Organisations is crucial to identify and manage cases in Aboriginal communities. Cutaneous diphtheria must be treated to reduce the risk of progression to respiratory disease in the affected individual and onward transmission to at-risk contacts. Surveillance must be strengthened through systematic WGS integrated with epidemiological investigations, to track outbreaks and monitor for emergence of antimicrobial resistance. Finally, improvements in access to housing, healthcare and education are needed to reduce the burden of diphtheria and other conditions by reducing poverty in Aboriginal communities [25].

## Conclusion

The diphtheria outbreak in the NT in 2025 and 2026 is occurring in a highly vaccinated population that experiences social and health inequity. Antibiotic therapy is crucial to interrupt transmission, and while vaccination and judicious use of DAT are key to preventing morbidity and mortality, improvements to housing must be prioritised.

## Data Availability

Sequence data used in this manuscript are available in the NCBI Sequence Read Archive (BioProject PRJNA1466032).

## †Erratum

At publication of this article, the entry 'Pseudomenbrane' in Table 1 mistakenly read 'Fever'. This has been corrected on 12 June 2026. We apologise for the mistake.

## Supplementary Data

107000 Supplement
