# Erratum for Euro Surveill. 2026;31(23)

**DOI:** 10.2807/1560-7917.ES.2026.31.24.260618e

**Published:** 2026-06-18

**Authors:** Eurosurveillance editorial team

**Affiliations:** 1 European Centre for Disease Prevention and Control (ECDC), Stockholm, Sweden

In the article ‘Diphtheria outbreak, Northern Territory of Australia, 2025 to 2026’ by Draper et al., published on 11 June 2026, the entry 'Pseudomenbrane' in Table 1 mistakenly read 'Fever'. This has been corrected on 12 June 2026. We apologise for the mistake. 

