# Supplementary material for: Diphtheria outbreak, Northern Territory of Australia, 2025 to 2026
Source: Euro Surveill. 2026 Jun 11;31(23):2600443. doi: 10.2807/1560-7917.ES.2026.31.23.2600443 (PMC13263650; doi:10.2807/1560-7917.ES.2026.31.23.2600443)
Supplement: Supplement [file 26-00443_DRAPER_Supplement.pdf]

This supplementary material is hosted by *Eurosurveillance* as supporting information alongside the article [Diphtheria outbreak, Northern Territory of Australia, 2025 to 2026], on behalf of the authors, who remain responsible for the accuracy and appropriateness of the content. The same standards for ethics, copyright, attributions and permissions as for the article apply. Supplements are not edited by *Eurosurveillance* and the journal is not responsible for the maintenance of any links or email addresses provided therein.

Supplementary Material

Laboratory Methods

Systematic testing for the *tox* gene in *Corynebacterium diphtheriae* isolates at Territory Pathology commenced on 24 February 2025, and this study continued from then until 30 April 2026.

Rayon-tipped swabs in semi-solid Amies medium were used to collect wound and throat swabs. Wound swabs were inoculated onto colistin-nalidixic acid (CNA) agar and MacConkey agar which were incubated at 37°C in air, and horse blood agar which was incubated 37°C in air and in 5% CO_2. Throat swabs were inoculated onto CNA agar and incubated anaerobically at 37°C. Where diphtheria was suspected, skin and throat swabs were inoculated onto Hoyles tellurite medium and incubated at 37°C in air. Cultures were examined after 24 and 48 hours' incubation (and at 72 hours for Hoyles plates). Colonies consistent with *Corynebacterium* species were identified using mass spectrometry using Vitek MS with version 3.3 of the knowledge base (bioMérieux, France), with a confidence score of 99.9% required for confirmation. Most of the cutaneous *C. diphtheriae* isolations were incidentally found on standard cultures, whereas most respiratory isolations were from Hoyles medium.

Antimicrobial susceptibility testing was done using gradient diffusion (E test) strips (bioMérieux, France), using a 0.5 McFarland standard on Mueller-Hinton (MH) with sheep blood or MH with horse blood and NAD (depending on media availability), incubated at 37°C in air and read at 18 hours.

DNA was extracted from colonies using the Roche MagNA Pure 24 total NA isolation kit (Roche diagnostics, Australia) using the optimised protocol for bacterial nucleic acid isolation (Pathogen 200). Quantitative polymerase chain reaction (qPCR) for detection of the *tox* gene was performed on the DNA extracts of *C. diphtheriae* isolates using primers, probe, and cycling conditions described previously [1]. Library preparation was conducted using the Illumina DNA library prep kit (Illumina, USA) according to manufacturer's instructions, and whole genome sequencing was done with an Illumina NextSeq 2000 using a 300 cycle P1 sequencing reagent kit (Illumina, USA).

Sequence data were quality filtered and trimmed using Trimmomatic v0.27 [2]. The genomes were assembled using SPAdes v3.14.0 [3], and assembly quality was assessed using QUAST v5 [4] and CheckM v1.2.3 [5]. Samples were considered to have passed quality control if N50 was >20,000 base pairs, total assembly length was within 10% of the 2.5 Mb expected genome size of *C. diphtheriae,* completeness was >90% and contamination was <5%. Samples which failed quality control on the first round of sequencing were sequenced a second time, and the data from each run was combined. For genomes passing quality control, multi-locus sequence typing was conducted with MLST v2.22.0 [6]. Abricate v1.2.0 [7] was used to compare samples to the VFDB v6.0 [8]

and RESfinder v4.0 [9] databases to determine the presence of virulence and antimicrobial resistance genes in the isolates. Confirmation of a complete *tox* gene was conducted by aligning trimmed read data to the NCTC13129 *tox* gene sequence (NC_002935.2:188979-190661) using BWA v0.7.19 [10], followed by variant calling using Bcftools v1.22 [11] and comparison NCBI core nucleotide database using ENTREZ v1.76 [12].

We used Snippy v4.6.0 [13] to conduct a core single nucleotide polymorphism (SNP) analysis of the samples in this study and publicly available ST381 genomes, including 21 from North Queensland, 3 with links to North Queensland, two from the Solomon Islands and one from Papua New Guinea [14–16]. The genome of strain CD38, an ST381 isolate collected from a cutaneous lesion in NSW in 2013 [17], was used as a reference. Recombination was identified and masked using Gubbins v3.4.1 [18], and SNP distances were calculated using snp-dists v1.2.0 [19].

A maximum likelihood phylogeny of the ST381 isolates was constructed using IQ-Tree2 v2.4.0 [20] with automated model selection and 5,000 bootstrap replicates, 1,000 replications of the Shimodaira–Hasegawa-like approximate likelihood ratio test, and with a minimum correlation coefficient set to 0.90. The phylogeny was visualised using iTOL v7.2.1[21].

In order to determine the time to most recent common ancestor between the genomes in this study and other ST381 genomes, we assessed temporal signal using root-to-tip regression implemented in TempEst v1.5.3 [22], using isolate sampling dates to evaluate the suitability of the data for molecular clock analysis. Time-scaled phylogenetic inference was performed using BactDating v1.1 [23] in R [24], applying a strict clock with gamma-distributed rate variation and incorporating isolate sampling dates as tip calibrations. A strict molecular clock was selected given the low genetic diversity observed within the ST381 lineage. Markov chain Monte Carlo (MCMC) chains were run for 20,000,000 generations sampled every 5,000 states with a burn-in of 10,000,000 iterations, with convergence assessed by visual inspection of trace plots and effective sample sizes using the coda R package [25]. The time to the most recent common ancestor (tMRCA) between Northern Territory isolates and those associated with the 2022 North Queensland outbreak was estimated from the posterior distribution, with uncertainty reported as 95% credible intervals.

References

1. Schuhegger R, Lindermayer M, Kugler R, Heesemann J, Busch U, Sing A. Detection of Toxigenic Corynebacterium diphtheriae and Corynebacterium ulcerans Strains by a Novel Real-Time PCR. J Clin Microbiol. 2008 Aug;46(8):2822–3. doi:10.1128/JCM.01010-08 PubMed PMID: 18550743; PubMed Central PMCID: PMC2519500.

2. Bolger AM, Lohse M, Usadel B. Trimmomatic: a flexible trimmer for Illumina sequence data. Bioinformatics. 2014 Aug 1;30(15):2114–20. doi:10.1093/bioinformatics/btu170

3. Bankevich A, Nurk S, Antipov D, Gurevich AA, Dvorkin M, Kulikov AS, et al. SPAdes: A New Genome Assembly Algorithm and Its Applications to Single-Cell Sequencing. Journal of Computational Biology. 2012 Apr 16;19(5):455–77. doi:10.1089/cmb.2012.0021

4. Gurevich A, Saveliev V, Vyahhi N, Tesler G. QUAST: quality assessment tool for genome assemblies. Bioinformatics. 2013 Apr 15;29(8):1072–5. doi:10.1093/bioinformatics/btt086 PubMed PMID: 23422339; PubMed Central PMCID: PMC3624806.

5. Parks DH, Imelfort M, Skennerton CT, Hugenholtz P, Tyson GW. CheckM: assessing the quality of microbial genomes recovered from isolates, single cells, and metagenomes. Genome Res. 2015 Jul;25(7):1043–55. doi:10.1101/gr.186072.114 PubMed PMID: 25977477; PubMed Central PMCID: PMC4484387.

6. Open-access bacterial population genomics: BIGSdb software, the PubMLST.org website and their applications - PMC [Internet]. [cited 2026 Jan 8]. Available from: https://pmc.ncbi.nlm.nih.gov/articles/PMC6192448/

7. tseemann. GitHub [Internet]. [cited 2026 Jan 28]. GitHub - tseemann/abricate: :mag_right: Mass screening of contigs for antimicrobial and virulence genes. Available from: https://github.com/tseemann/abricate

8. Chen L, Yang J, Yu J, Yao Z, Sun L, Shen Y, et al. VFDB: a reference database for bacterial virulence factors. Nucleic Acids Res. 2005 Jan 1;33(Database issue):D325-328. doi:10.1093/nar/gki008 PubMed PMID: 15608208; PubMed Central PMCID: PMC539962.

9. Florensa AF, Kaas RS, Clausen PTLC, Aytan-Aktug D, Aarestrup FM. ResFinder – an open online resource for identification of antimicrobial resistance genes in next-generation sequencing data and prediction of phenotypes from genotypes. Microb Genom. 2022 Jan 24;8(1):000748. doi:10.1099/mgen.0.000748 PubMed PMID: 35072601; PubMed Central PMCID: PMC8914360.

10. Li H. Aligning sequence reads, clone sequences and assembly contigs with BWA-MEM. arXiv:13033997 [q-bio] [Internet]. 2013 May 26 [cited 2020 Jan 4]. Available from: http://arxiv.org/abs/1303.3997

11. Danecek P, Bonfield JK, Liddle J, Marshall J, Ohan V, Pollard MO, et al. Twelve years of SAMtools and BCFtools. GigaScience. 2021 Feb 1;10(2):giab008. doi:10.1093/gigascience/giab008

12. Kans J. Entrez Direct: E-utilities on the Unix Command Line. Entrez Programming Utilities Help [Internet] [Internet]. National Center for Biotechnology Information

(US); 2021 [cited 2021 May 7]. Available from: https://www.ncbi.nlm.nih.gov/books/NBK179288/

13. Seemann T. tseemann/snippy [Perl] [Internet]. 2025 [cited 2025 Oct 9]. Available from: https://github.com/tseemann/snippy

14. Graham RMA, Rathnayake IU, Sandhu S, Bhandari M, Taunton C, Fisher V, et al. Genomic analysis of an outbreak of toxin gene bearing Corynebacterium diphtheriae in Northern Queensland, Australia reveals high level of genetic similarity. Epidemiol Infect. 2023 May 22;151:e92. doi:10.1017/S0950268823000699 PubMed PMID: 37212056; PubMed Central PMCID: PMC10311691.

15. Doyle CJ, Mazins A, Graham RMA, Fang NX, Smith HV, Jennison AV. Sequence Analysis of Toxin Gene–Bearing Corynebacterium diphtheriae Strains, Australia. Emerg Infect Dis. 2017 Jan;23(1):105–7. doi:10.3201/eid2301.160584 PubMed PMID: 27983494; PubMed Central PMCID: PMC5176206.

16. Simpson A, Douglas P, Draper J, Sintchenko V, Cutcher Z, Ashton D. Respiratory diphtheria in the time of Omicron. Commun Dis Intell (2018). 2024 Aug 21;48. doi:10.33321/cdi.2024.48.41 PubMed PMID: 39165018.

17. Timms VJ, Nguyen T, Crighton T, Yuen M, Sintchenko V. Genome-wide comparison of Corynebacterium diphtheriae isolates from Australia identifies differences in the Pan-genomes between respiratory and cutaneous strains. BMC Genomics. 2018 Dec 4;19(1):869. doi:10.1186/s12864-018-5147-2

18. Croucher NJ, Page AJ, Connor TR, Delaney AJ, Keane JA, Bentley SD, et al. Rapid phylogenetic analysis of large samples of recombinant bacterial whole genome sequences using Gubbins. Nucleic Acids Res. 2015 Feb 18;43(3):e15. doi:10.1093/nar/gku1196

19. Seemann T. Source code for snp-dists software [Internet]. Zenodo; 2018 [cited 2026 Jan 28]. Available from: https://zenodo.org/records/1411986 doi:10.5281/zenodo.1411986

20. Minh BQ, Schmidt HA, Chernomor O, Schrempf D, Woodhams MD, von Haeseler A, et al. IQ-TREE 2: New Models and Efficient Methods for Phylogenetic Inference in the Genomic Era. Mol Biol Evol. 2020 May 1;37(5):1530–4. doi:10.1093/molbev/msaa015

21. Letunic I, Bork P. Interactive Tree of Life (iTOL) v6: recent updates to the phylogenetic tree display and annotation tool. Nucleic Acids Res. 2024 Jul 5;52(W1):W78–82. doi:10.1093/nar/gkae268

22. Rambaut A, Lam TT, Max Carvalho L, Pybus OG. Exploring the temporal structure of heterochronous sequences using TempEst (formerly Path-O-Gen). Virus Evol. 2016 Jan 1;2(1):vew007. doi:10.1093/ve/vew007

23. Didelot X, Croucher NJ, Bentley SD, Harris SR, Wilson DJ. Bayesian inference of ancestral dates on bacterial phylogenetic trees. Nucleic Acids Res. 2018 Dec 14;46(22):e134. doi:10.1093/nar/gky783

24. Team RC. R language definition. Vienna, Austria: R foundation for statistical computing. 2000;3(1):116.

25. Plummer M, Best N, Cowles K, Vines K. CODA: convergence diagnosis and output analysis for MCMC. R news. 2006;6(1):7–11.
